# RETRACTION: The Preventive Effect of *Lactobacillus plantarum* ZS62 on DSS‐Induced IBD by Regulating Oxidative Stress and the Immune Response

**DOI:** 10.1155/omcl/9802420

**Published:** 2026-10-28

**Authors:** Oxidative Medicine and Cellular Longevity

RETRACTION: Y. Pan, Y. Ning, J. Hu, Z. Wang, X. Chen, and X. Zhao, “The Preventive Effect of *Lactobacillus plantarum* ZS62 on DSS‐Induced IBD by Regulating Oxidative Stress and the Immune Response,” *Oxidative Medicine and Cellular Longevity* 2021, no. 1 (2021): 9416794, https://doi.org/10.1155/2021/9416794.

The above article, published online on 27 October 2021 in Wiley Online Library (wileyonlinelibrary.com), has been retracted by agreement between the Journal Editor‐in‐Chief, Jeannette Vasquez Vivar; and John Wiley & Sons Ltd. The retraction has been agreed after an investigation into concerns raised by *Indigofera tanganyikensis* on PubPeer [1].

Specifically:

• Figure 2b shares unexpected similarities with Figure 1a in [2], which shares at least one author;
• The western blots in Figures 6 and 7 share unexpected similarities.

The authors had contacted the journal to request a correction to Figure 2b, and stated that the similarities between the western blots were due to stripping and re‐probing the same membrane using Thermo Scientific Restore Stripping Buffer, Cat. No. 21059. They also stated that the colon image duplication was due to data management errors, and that the paper’s conclusions are fully supported by independent replicate data from the original study period.

However, the journal and Editor found their reasoning, and the data provided, to be insufficient. As such, we are no longer confident in the results and conclusions. Therefore, the article must be retracted.

The authors disagree with this retraction.
